# Activated low-density granulocytes in peripheral and intervillous blood and neutrophil inflammation in placentas from SLE pregnancies

**DOI:** 10.1136/lupus-2020-000463

**Published:** 2021-03-07

**Authors:** Marit Stockfelt, Gunilla Larsson, Hanna Engström, Henri Puttonen, Henrik Zetterberg, Kaj Blennow, Christopher Sjöwall, Helena Strevens, Andreas Jönsen, Anders A Bengtsson, Maria Majczuk Sennström, Agneta Zickert, Elisabet Svenungsson, Iva Gunnarsson, Estelle Trysberg, Bo Jacobsson, Anna-Karin Hultgård Ekwall, Karin Christenson, Johan Bylund, Mattias N D Svensson, Anna-Carin Lundell

**Affiliations:** 1 Dept of Rheumatology and Inflammation Research, Institute of Medicine, Sahlgrenska Academy at the University of Gothenburg, Gothenburg, Sweden; 2 Department of Pathology, Sahlgrenska University Hospital, Goteborg, Sweden; 3 Department of Psychiatry and Neurochemistry, Institute of Neuroscience and Physiology, Sahlgrenska Academy at University of Gothenburg, Gothenburg, Sweden; 4 Clinical Neurochemistry Laboratory, Sahlgrenska University Hospital, Mölndal, Sweden; 5 UK Dementia Research, Institute at UCL, London, UK; 6 Department of Neurodegenerative Disease, UCL Institute of Neurology, London, UK; 7 Division of Inflammation and Infection, Department of Biomedical and Clinical Sciences, Linköping University, Linköping, Sweden; 8 Department of Obstetrics and Gynecology, Institute of Clinical Sciences, Skåne University Hospital, Lund, Sweden; 9 Department of Clinical Sciences, Rheumatology, Lund University, Lund, Sweden; 10 Department of Womens and Childrens Health, Division for Obstetrics and Gynecology, Karolinska University Hospital, Karolinska Institute, Stockholm, Sweden; 11 Division of Rheumatology, Department of Medicine, Karolinska Institutet, Karolinska University Hospital, Stockholm, Sweden; 12 Department of Obstetrics and Gynecology, Institute of Clinical Sciences, Sahlgrenska Academy, University of Gothenburg, Goteborg, Sweden; 13 Region Västra Götaland, Sahlgrenska University Hospital, Department of Obstetrics and Gynecology, Gothenburg, Sweden; 14 Department of Genetics and Bioinformatics, Domain of Health Data and Digitalisation, Institute of Public Health, Oslo, Norway; 15 Department of Oral Microbiology and Immunology, Institute of Odontology, Sahlgrenska Academy at the University of Gothenburg, Goteborg, Sweden

**Keywords:** lupus erythematosus, systemic, chemokines, fibroblasts

## Abstract

**Objective:**

Women with SLE face an increased risk of adverse pregnancy outcomes compared with healthy women, but the underlying immunological mechanisms are unknown. Given the recognised association of neutrophil activation with SLE pathogenesis, we examined whether there is increased neutrophil activation and inflammation in blood and placenta in SLE relative to healthy pregnancy.

**Methods:**

At delivery, peripheral blood, maternal-derived intervillous blood and placentas were collected from 12 SLE and 10 healthy control pregnancies. The proportion of low-density granulocytes (LDGs) and the activation status of LDG and normal-density granulocytes were examined with flow cytometry. The chemokines CXCL8 and CXCL1 were quantified with a cytometric bead-based assay and interferon alpha (IFNα) protein levels with a Simoa method. IFNα-stimulated maternal-derived decidual stromal cells were examined for *CXCL8* gene expression with qPCR. A pathologist, blinded to the patient background, examined all placentas.

**Results:**

Women with SLE had significantly higher proportions of LDG in peripheral blood compared with controls (p=0.02), and LDG in both peripheral and intervillous blood were more activated in SLE relative to healthy pregnancies (peripheral blood: p=0.002 and intervillous blood: p=0.05). There were higher levels of CXCL8 and CXCL1 in intervillous compared with peripheral blood in women with SLE (p=0.004 and p=<0.0001, respectively) but not in controls. In SLE pregnancy, IFNα was detectable in 6 out of 10 intervillous blood samples but only in one control. Stimulation with IFNα upregulated *CXCL8* gene expression in decidual stromal cells from both SLE and healthy pregnancy. Histological chorioamnionitis was present in 6 out of 12 placentas from women with SLE and in 1 out of 10 controls.

**Conclusions:**

In women with SLE, locally produced chemokines in the placenta are increased and may attract and activate neutrophils. This in turn could contribute to placental inflammation and dysfunction and increased risk of placenta-related pregnancy complications.

**Key messages**

**What is already known about this subject?**

- Women with SLE face an increased risk of adverse pregnancy outcomes compared with healthy women, but the underlying immunological mechanisms are unknown.

**What does this study add?**

- Women with SLE have higher circulating proportions and increased activation of low-density granulocytes at delivery compared with healthy women.
- The levels of the neutrophil-attracting and activating chemokines CXCL8 and CXCL1 are higher in maternal-derived intervillous blood relative to peripheral blood in women with SLE.
- Maternal-derived decidual stromal cells are a cellular source of CXCL8 and CXCL1.
- Increased neutrophil activation may contribute to placental inflammation in pregnant women with SLE.

## Introduction

SLE is a chronic inflammatory disease that affects women nine times more frequently than men, and disease onset is common during fertile ages.^1^ SLE is a heterogeneous disease that commonly affects the skin and joints but may also involve vital organs such as the kidneys and the central nervous system. Although modern disease surveillance and treatment has improved pregnancy outcome,^2^ women with SLE still face a higher risk of pregnancy complications including pre-eclampsia, preterm birth and stillbirth.^3 4^ In a recent Swedish study, 56% of SLE pregnancies were complicated with at least one adverse pregnancy outcome, including caesarean section.^5^ Our understanding of the immunological mechanisms preceding these pregnancy complications and our ability to predict and treat them are limited.

The placental basal plate is a site of interaction between maternal and fetal tissues. The maternal-derived decidua basalis is composed of a stromal network that harbours maternal-derived immune cells and spiral arteries, which deposit oxygenated blood and nutrients into the intervillous space. In healthy term pregnancy, intervillous blood display distinct immune cell and chemokine profiles compared with peripheral blood with higher proportions of effector memory T cells and MAIT cells as well as the chemokines CXCL10 and MIF in intervillous blood.^6^ Yet, intervillous blood from women with SLE is unexplored.

In recent years, neutrophils have emerged as key effector cells in the pathogenesis of SLE.^7^ In SLE pregnancy, a neutrophil transcriptional profile is enriched in peripheral blood compared with healthy pregnant women.^8^ One study also reported more neutrophils and increased neutrophil extracellular traps (NETs) in the placental intervillous space from SLE compared with healthy pregnancy.^9^ First discovered in SLE, a subset of low-density granulocytes (LDGs) colocalise with mononuclear cells after gradient centrifugation.^10^ In SLE, LDG display spontaneous NET formation and CXCL8 secretion, a chemokine that attracts and activates neutrophils.^11 12^ Neither the presence of LDG nor the priming of LDG havs been studied in peripheral or intervillous blood of pregnant women with SLE.

The presence of neutrophils, which are normally absent in the placental fetal-derived membranes, can be examined through placental histological examination. Histological acute chorioamnionitis is characterised by infiltration of neutrophils into the chorioamniotic membranes and may also occur during non-infectious inflammation,^13 14^ but the cause for this phenomenon is unclear. Few studies have compared the placental histological pattern in women with SLE to that of healthy women.

In SLE, about 50%–75% of adult patients display overexpression of type I interferon (IFN)-inducible genes, the IFN signature.^15^ It has been indicated that the IFN signature remains prominent in women with complicated SLE pregnancy, while this signature is downregulated compared with baseline in women with a healthy SLE pregnancy.^8^ Furthermore, expression of the IFNα-responsive gene *MX1* is increased in women with SLE who develop pre-eclampsia compared with women with SLE who do not.^16^ Protein levels of IFNα have been difficult to quantify, but with a novel ultrasensitive digital ELISA, based on single molecule array (Simoa) technology, increased IFNα serum levels have been detected in patients with SLE compared with controls and relate to disease activity^17^ and risk of relapse.^18^ IFNα levels in pregnant women with SLE remains to be examined.

The aim of the current study was to investigate the presence and activation status of neutrophil subsets in paired samples of peripheral and intervillous blood and the potential for neutrophil recruitment to the placenta in women with SLE and healthy pregnancy.

## Methods

### Patients and healthy controls

Pregnant women with SLE (n=12) were recruited at rheumatology clinics in Gothenburg (Sahlgrenska University Hospital), Stockholm (Karolinska University Hospital) and Lund (Skåne University Hospital), and healthy age-matched pregnant women (n=10) were recruited at one antenatal clinic in Gothenburg (n=10). All women with SLE fulfilled the 1997 American College of Rheumatology (ACR) and/or the 2012 Systemic Lupus International Collaborating Clinics (SLICC) classification criteria.^19 20^ Disease activity was evaluated with Systemic Lupus Disease Activity Index 2000 (SLEDAI-2K) at some time point during pregnancy (weeks 7–34).^21^ Exclusion criteria were inability to understand the patient information and, for women with SLE, presence of other serious diseases and treatment with anti-CD20 or anti-BAFF antibodies within a period of 12 months before inclusion. Adverse pregnancy outcomes were defined as one or more of the following: (1) pre-eclampsia at any time according to 2019 national guidelines from the Swedish Society of Obstetrics and Gynecology, (2) preterm delivery <37th gestational week and (3) small for gestational age (SGA), with a birth weight below mean −2SD, calculation based on birth weight, gender and gestational age.^22^ All participants gave informed consent and the Ethics Committe of Gothenburg approved the study protocol (Dnr 404-18).

### Isolation of plasma and immune cells from peripheral and intervillous blood

Peripheral blood samples were collected in heparin tubes after admission to the delivery ward, and the placenta was collected after delivery. Within 24 hours after delivery, handling of blood samples and placentas was initiated at our laboratory in Gothenburg. The placenta was kept cold during transport, and blood samples were kept at room temperature. The placenta was lifted so that blood from the intervillous space could drip down onto a sterile petri dish and then quickly transferred to heparin tubes. Peripheral and intervillous blood was diluted 1:1 with phosphate-buffered saline (PBS), layered on Ficoll-Paque plus (GE Healthcare, Uppsala, Sweden) and centrifuged (900× g, 20 min, without brake). After centrifugation, mononuclear cells, which are located above the Ficoll layer, including LDGs if present, were collected as well as normal-density granulocytes (NDGs) that end up in the pellet. Plasma was saved and frozen (−80°C) until use. Due to small volume of blood samples from some individuals, all immunological assays could not be applied. In addition, when delivery occurred during weekends, a partial assay protocol for blood samples was used.

### Isolation and expansion of stromal cells from maternal-derived decidua basalis

Tissue samples from six women with SLE and six healthy controls were dissected from maternal-derived decidua basalis and washed thoroughly with PBS. Tissue pieces were minced and enzymatically digested with Liberase (0.1 U/mL, Sigma-Aldrich, St. Louis, Missouri, USA) mixed with Dulbecco’s modified Eagles medium (DMEM; HyClone, Utah, USA) in 5% CO_2_ at 37°C for 60 min. Tissues and cells were washed and cultured in flasks with DMEM containing 10% fetal bovine serum (FBS; HyClone) and gentamicin (Sigma-Aldrich) (complete DMEM). When 90%–95% confluent, cells were harvested with trypsin/EDTA (Thermo Fisher Scientific, Waltham, Massachusetts, USA), washed with complete DMEM medium and seeded in new flasks. Cells were harvested after first passage and frozen in FBS with 7.5% dimethylsulfoxide (DMSO).

### Flow cytometry

To analyse total granulocyte counts in peripheral and intervillous blood, the Trucount assay was applied. In brief, antihuman monoclonal PerCP-conjugated CD45 antibodies (clone 2D1, BD Biosciences, New Jersey, USA) and whole blood were added to BD Trucount tubes (BD Biosciences) and incubated for 15 min. Red blood cells were lysed using BD FACS Lysis Solution (BD Biosciences). Analysis of the proportion of LDG among mononuclear cells in peripheral and intervillous blood was based on CD45 expression and side scatter characteristics. NDG and LDG activation was examined by CD62L and CD11b expressions. In brief, red blood cells present among NDG cells were lysed by a short incubation with dH_2_O, followed by addition of PBS (with 25 g NaCl/L), which was repeated twice. NDG and mononuclear cells including LDG were then stained with the following antihuman monoclonal antibodies: PE-conjugated CD62L (clone SK11, BD Biosciences), APC-conjugated CD11b (clone D12, BD Biosciences) and FITC-conjugated CD45 (clone 2D1, Biolegend, San Diego, California, USA) for 20 min. To exclude dead cells, 7-aminoactinomycin D (BD Biosciences) was used. To verify a stroma phenotype, decidual stromal cells were stained with the following antihuman monoclonal antibodies: FITC-conjugated CD90 (clone 5E10, BD Biosciences), Bv421-conjugated CD105 (clone 266, BD Biosciences), PE-conjugated CD31 (clone WM59, BD Biosciences) and APC-conjugated CD45 (clone NZ-1.3, BD Biosciences). All samples were acquired in a FACSVerse equipped with FACSuite Software (BD Biosciences) and analysed with FlowJo Software (TreeStar, Ashland, Oregon, USA).

### Quantification of CXCL8, CXCL5, CXCL1 and IFNα in plasma

The CXCL8, CXCL5 and CXCL1 levels in peripheral and intervillous blood were quantified with flow cytometry bead-based immunoassay (LEGENDplex, Biolegend) according to the manufacturer’s instructions. Data were acquired in a FACSVerse equipped with FACSuite Software and analysed with FCAP array software (SoftFlow, Pecs, Hungary). The level of IFNα in plasma from peripheral and intervillous blood was quantified with Simoa on an HD-1 Analyzer (Quanterix, Billerica, Massachusetts, USA).

### Quantitative PCR (qPCR)

To examine *CXCL8*, *CXCL1* and *MX1* gene expression in decidual stromal cells in passage 1, cells were thawed and cultured in complete DMEM in flasks (7.5×10^4^ cells/mL) for 4 days. Thereafter, cells were washed with PBS and cultured overnight in DMEM with 0.1% FBS (starvation medium). Next, cells were washed with PBS and cultured with or without IFNα (1 ng/mL; PBL Biomedical Laboratories, Piscataway, New Jersey, USA) for 24 hours in starvation medium. The cells were washed with PBS, after which cells were lysed by adding 350 µL Buffer RLT (Qiagen, Hilden, Germany) with beta-mercaptoethanol (1:100). RNA was extracted from lysates using the RNeasy Kit (Qiagen) and used to generate complementary DNA (cDNA) with the iScript cDNA synthesis kit (Bio-Rad, Hercules, California, USA). qPCR was performed on a ViiA7 Real-Time PCR System (Applied Biosystems, Waltham, Massachusetts, USA) with SsoAdvanced Universal SYBR Green Supermix (Bio-Rad). Primer assays for *CXCL8*, *CXCL1*, *MX1* and *GAPDH* were obtained from Sigma-Aldrich. Each reaction was normalised to the expression levels of the housekeeping gene *GAPDH*. Results are presented as fold change (relative quantification) compared with the expression level in healthy control samples with the ΔΔ*C*t method.

### Placenta histology

Placenta specimens of 12 women with SLE and 10 healthy controls were assessed for the presence of histological acute chorioamnionitis and funisitis by a perinatal pathologist. The morphological evaluation and histological sampling were performed in accordance with recommendations of the Swedish Society of Pathology and the Royal College of Pathologists.^23^ In brief, tissue samples were obtained from full-thickness sections of central placental parenchyma including the fetal and maternal surfaces, roll of external fetal membranes and the umbilical cord. Histological acute chorioamnionitis was defined as neutrophil infiltration of chorionic plate and amnion, while funisitis was defined as neutrophilic infiltration of fetal vessel walls and the surrounding connective tissue.

### Statistical analysis

Mann-Whitney U test was used to compare granulocyte numbers, activation markers on granulocyte subsets and proportions of LDGs in peripheral and intervillous blood from women with SLE and healthy controls. Kruskal-Wallis test followed by Dunn’s multiple comparison test were used to compare CXCL8, CXCL5, CXCL1 and IFNα levels in peripheral or intervillous blood plasma from women with SLE and healthy controls (GraphPad Prism software, La Jolla, California, USA). A p value of ≤0.05 was considered statistically significant (*p≤0.05, **p≤0.01, ***p≤0.001 and ****p≤0.0001).

## Results

### Baseline characteristics and prevalence of adverse pregnancy outcomes

Peripheral blood and placentas from 12 nulliparous women with SLE and from 10 nulliparous healthy women were collected at the time of delivery. Among women with SLE, one woman gave birth to twins (two placentas). Baseline characteristics of the included patients with SLE are presented in table 1. All fulfilled the ACR and/or SLICC classification criteria,^19 20^ and all had ANA. Five of the women were ever positive for antiphospholipid antibodies, but none fulfilled the criteria for antiphospholipid syndrome. All but one had immunosuppressant medication (table 1). Any adverse pregnancy outcome (APO), including preterm delivery, pre-eclampsia or SGA, occurred in 17% (2 out of 12) of the women with SLE and in 20% (2 out of 10) of the healthy controls (table 2). None of the women had confirmed COVID-19 at delivery.

**Table 1:** Baseline characteristics of the included patients with SLE

Characteristics	Women with SLE (n=12)
ACR criteria*	
Malar rash	3 (25)
Discoid rash	2 (16)
Photosensitivity	4 (33)
Oral ulcers	3 (25)
Arthritis	10 (83)
Serositis	1 (8.3)
Renal disorder	7 (58)
Neurological disorder	0 (0)
Haematological disorder	6 (50)
Immunological disorder	9 (75)
ANA	12 (100)
Disease duration at inclusion (years)†	8.5 (0.5–15)
SLEDAI-2K score during pregnancy†‡	1 (0–2)
Ever anti-dsDNA*	10 (83)
Ever antiphospholipid antibodies*§	5 (42)
Antiphospholipid syndrome*	0 (0)
Medication at pregnancy onset*	
Hydroxychloroquine	11 (92)
Low molecular weight heparin¶	6 (50)
Acetylsalicylic acid¶	12 (100)
Any immunosuppressant medication**	11 (92)
Azatioprin	5 (42)
Prednisone	5†† (42)

*Data presented as n (%).

†Data presented as median (range).

‡Missing data for four participants.

§Including anti-β2-glycoprotein 1 antibody (IgA, IgG and IgM), anticardiolipin antibody (IgA, IgG and IgM) and lupus anticoagulans (dRVVT).

¶LMWH in four patients at pregnancy start and an additional two patients during pregnancy. Acetylsalicylic acid in nine patients at pregnancy start and an additional three patients during pregnancy.

**Including azatioprin and prednisone.

††Dose range 2.5–5.0 mg/day.

ACR, American College of Rheumatology; SLEDAI-2K, Systemic Lupus Disease Activity Index 2000.

**Table 2:** Demographic data

	Women with SLE (n=12)	Healthy women (n=10)
Age at inclusion*	30 (19–40)	26.5 (18–35)
Smoking at start of pregnancy†	0 (0)	0 (0)
Folic acid at start of pregnancy†	10 (83)	10 (100)
Gestational age, days*	279 (181–287)	282 (269–294)
Trimmed placental weight, g†‡	412 (257.4–492.8)	453 (333.3–590.0)
Birth weight of child, g†‡	3580 (2915–4190)	3507.5 (3115–4270)
Induced delivery	6 (50)	4 (40)
Elective caesarean section	1 (8.3)	0 (0)
Acute caesarean section	1 (8.3)	0 (0)
Adverse pregnancy outcome (APO)†		
Any APO	2 (17)	2 (20)
Preterm delivery§	1 (8.3)	0 (0)
Pre-eclampsia	1 (8.3)	2 (20)
Small for gestational age‡¶	0 (0)	0 (0)

*Data presented as median (range).

†Data presented as n (%).

‡Data from twin birth in the SLE group not included.

§<37th gestational week.

¶Defined according to 22.

APO, adverse pregnancy outcome.

### The proportions of LDGs and their activation status are higher in peripheral blood from women with SLE compared with healthy controls

**Figure 1:**
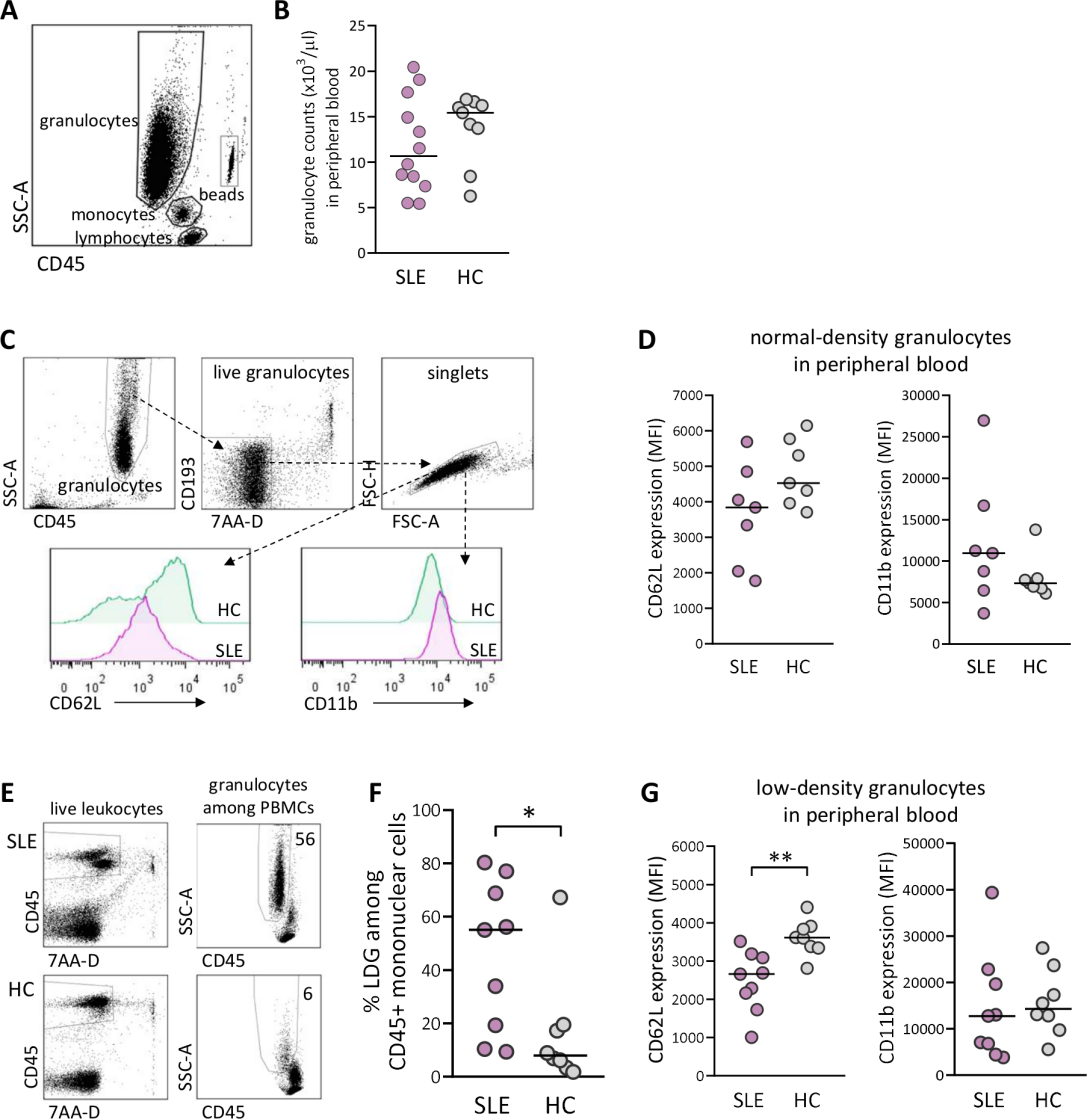
Elevated proportions and increased priming of low-density granulocytes (LDGs) in peripheral blood in women with SLE compared with healthy women. (A) Gating strategy for granulocytes in peripheral whole blood. (B) Granulocyte count in peripheral blood in women with SLE compared with healthy controls (HCs). (C) Gating strategy for live singlet normal-density granulocytes (NDGs) in peripheral blood and representative histograms of CD62L and CD11b expression within the NDG subset in one woman with SLE and one HC. (D) Expression of CD62L and CD11b on NDG in women with SLE relative to HC. (E) Representative gating strategy for live singlet LDGs among CD45^+^ mononuclear cells from one woman with SLE and one HC. (F) Percentage of LDG in women with SLE compared with HC. (G) Expression of CD62L and CD11b on LDG in women with SLE relative to HC. Horizontal bars indicate medians, and each symbol represents one individual.*P≤0.05 and **p≤0.01, Mann-Whitney U test. MFI, mean fluorescence intensity; PBMCs, peripheral blood mononuclear cells.

At delivery, there was no difference in total granulocyte number in peripheral blood between SLE and healthy pregnancy (figure 1A, B). As granulocytes can be subdivided into NDGs and LDGs, we examined these subsets with regard to the activation markers CD62L, which is shed on activation, and CD11b, which is upregulated on activation (gating strategy for NDG and activation markers in figure 1C). There was a trend for lower expression of CD62L and higher expression of CD11b on NDG in women with SLE compared with healthy women, but the differences were not statistically significant (figure 1D). The gating strategy for proportions of LDG among CD45^+^ mononuclear cells is presented in figure 1E. There was a large interindividual variation in the fraction of LDG in peripheral blood from women with SLE, and the median value was higher than in healthy women (p=0.02; figure 1F). Furthermore, SLE LDG displayed increased activation as demonstrated by significantly lower CD62L expression compared with LDG from controls (p=0.002; figure 1G). Thus, women with SLE have higher circulating proportions and increased priming of LDG at delivery compared with healthy women.

### Increased activation of LDGs in maternal-derived intervillous blood in women with SLE compared with healthy controls

**Figure 2:**
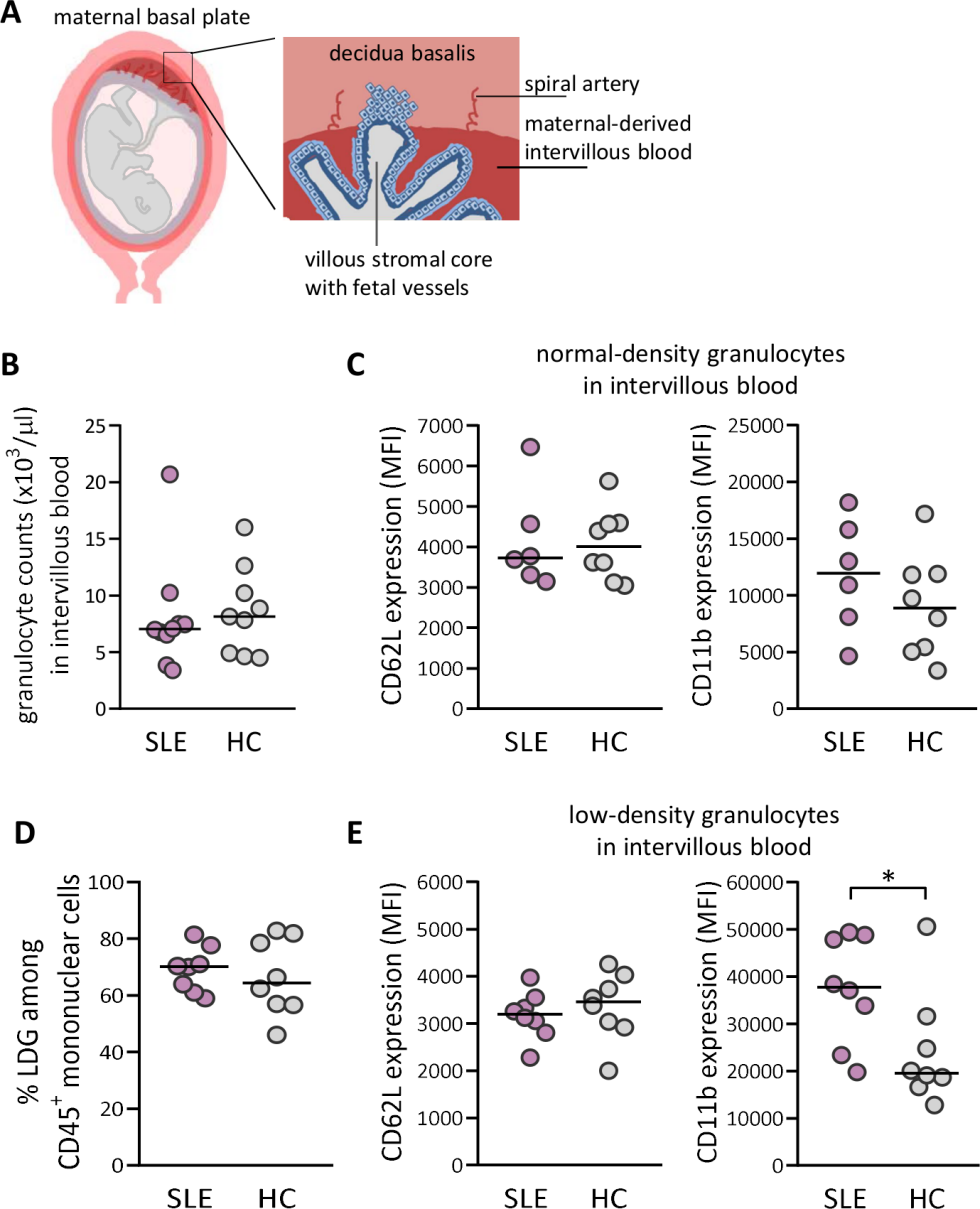
Increased priming of low-density granulocytes (LDGs) in maternal-derived intervillous blood in women with SLE compared with healthy women. (A) Schematic illustrations made by A-C Lundell that depict decidua basalis with spiral arteries and maternal-derived intervillous blood in relation to fetal villous tissue, which is modified from ref 39. (B) Granulocyte count in intervillous whole blood in women with SLE compared with healthy controls (HCs). (C) Expression of CD62L and CD11b on normal-density granulocytes in women with SLE relative to HC. (D) Percentage of LDGs in women with SLE compared with HC. (E) Expression of CD62L and CD11b on LDG in women with SLE compared with HC. Horizontal bars indicate medians, and each symbol represents one individual. *P≤0.05, Mann-Whitney U test.

The maternal-derived decidua harbours spiral arteries that deposit blood into the intervillous space (schematically presented in figure 2A). We next examined granulocyte numbers and granulocyte activation in intervillous blood from both SLE and healthy pregnancies (representative flow cytometry plots for intervillous blood are presented in online supplemental figure 1). Similar to peripheral blood, there was no difference in granulocyte number or in the activation status of NDG in intervillous blood between women with SLE and healthy women (figure 2B, C). In contrast to peripheral blood, there was no difference in proportions of LDG in intervillous blood between SLE and healthy pregnancy (figure 2D). However, LDG from women with SLE displayed increased activation as demonstrated by higher CD11b expression relative to those in healthy controls (p=0.05; figure 2E). In SLE, LDG in intervillous blood were more activated than in peripheral blood based on CD11b expression (p=0.003, online supplemental figure 2). Subgroup statistical analysis of proportions of LDG and activation status with respect to APO could not be performed due to low number of individuals (online supplemental figure 3A–C and 4A–C, respectively). Thus, LDG in both peripheral and intervillous blood from SLE pregnancy display increased activation compared with LDG in healthy pregnancy.

10.1136/lupus-2020-000463.supp1 Supplementary data

### Higher levels of neutrophil-attracting and activating chemokines in intervillous compared with peripheral blood in SLE pregnancy

**Figure 3:**
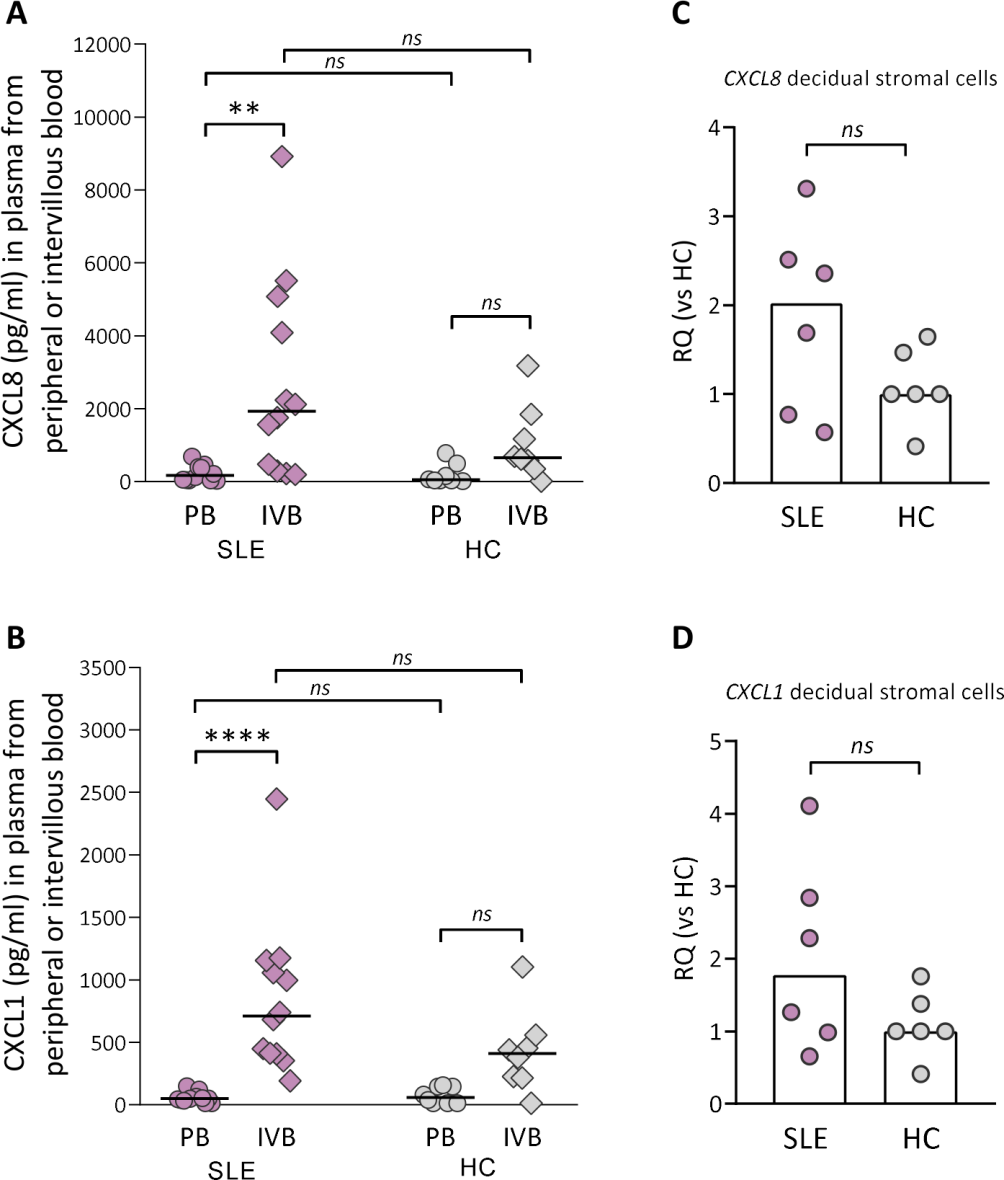
Neutrophil-attracting chemokine gradient between IVB and PB in women with SLE. Levels of CXCL8 (A) and CXCL1 (B) were compared in plasma obtained from peripheral or intervillous blood at delivery in SLE and healthy pregnancies (healthy control (HC)). Horizontal bars indicate medians, and each symbol represents one individual. **P≤0.01 and ****p≤0.0001, Kruskal-Wallis followed by Dunn’s multiple comparisons test. Quantitative PCR analysis of *CXCL8* (C) and *CXCL1* (D) gene expression in decidual stromal cells from women with SLE compared with HCs. Scatter plot bars display median, and each symbol represents one individual (Mann-Whitney U test). IVB, intervillous blood; PB, peripheral blood; RQ, relative quantification.

Although there was no difference in granulocyte counts in intervillous blood between SLE and healthy pregnancy, granulocytes may have been attracted to the placenta and migrated into the tissue. Therefore, we examined if there was a gradient in levels of CXCL8, CXCL5 and CXCL1, all neutrophil-attracting and activating chemokines, in intervillous relative to peripheral blood plasma from women with SLE and healthy controls. There was a large interindividual variation in the levels of CXCL8 and CXCL1 in intervillous blood from women with SLE, and the concentrations of both chemokines were higher compared with those in peripheral blood (p=0.004 and p≤0.0001, respectively; figure 3A, B). In healthy women, there were no significant differences in either CXCL8 or CXCL1 levels between intervillous and peripheral blood (figure 3A, B). The concentrations of CXCL8 or CXCL1 did not differ between women with SLE and controls either in peripheral or in intervillous blood (figure 3A, B). In contrast to CXCL8 and CXCL1, there was no difference in CXCL5 levels between intervillous and peripheral blood neither in women with SLE nor healthy controls (online supplemental figure 5). Given that stromal cells are important producers of chemokines, we examined the *CXCL8* and *CXCL1* gene expression in maternal-derived decidual stromal cells. *CXCL8* and *CXCL1* gene expression was upregulated in decidual stromal cells from three out of six of the women with SLE compared with controls (figure 3C, D, p=0.23 and p=0.39, respectively), which corresponds to the interindividual variation in CXCL8 and CXCL1 levels in intervillous blood. In both groups, decidual stromal cells expressed a homogenous phenotype with respect to expression of CD90 and CD105 used to define human stromal cells^24^ and were negative for the endothelial cell marker CD31 and the leucocyte marker CD45 (online supplemental figure 6). These data suggest that decidual stromal cells are a cellular source of CXCL8 and CXCL1, which may contribute to increased attraction and activation of neutrophils in pregnant women with SLE.

### IFNα protein is present in intervillous blood from SLE pregnancy and upregulates *CXCL8* gene expression in decidual stromal cells

**Figure 4:**
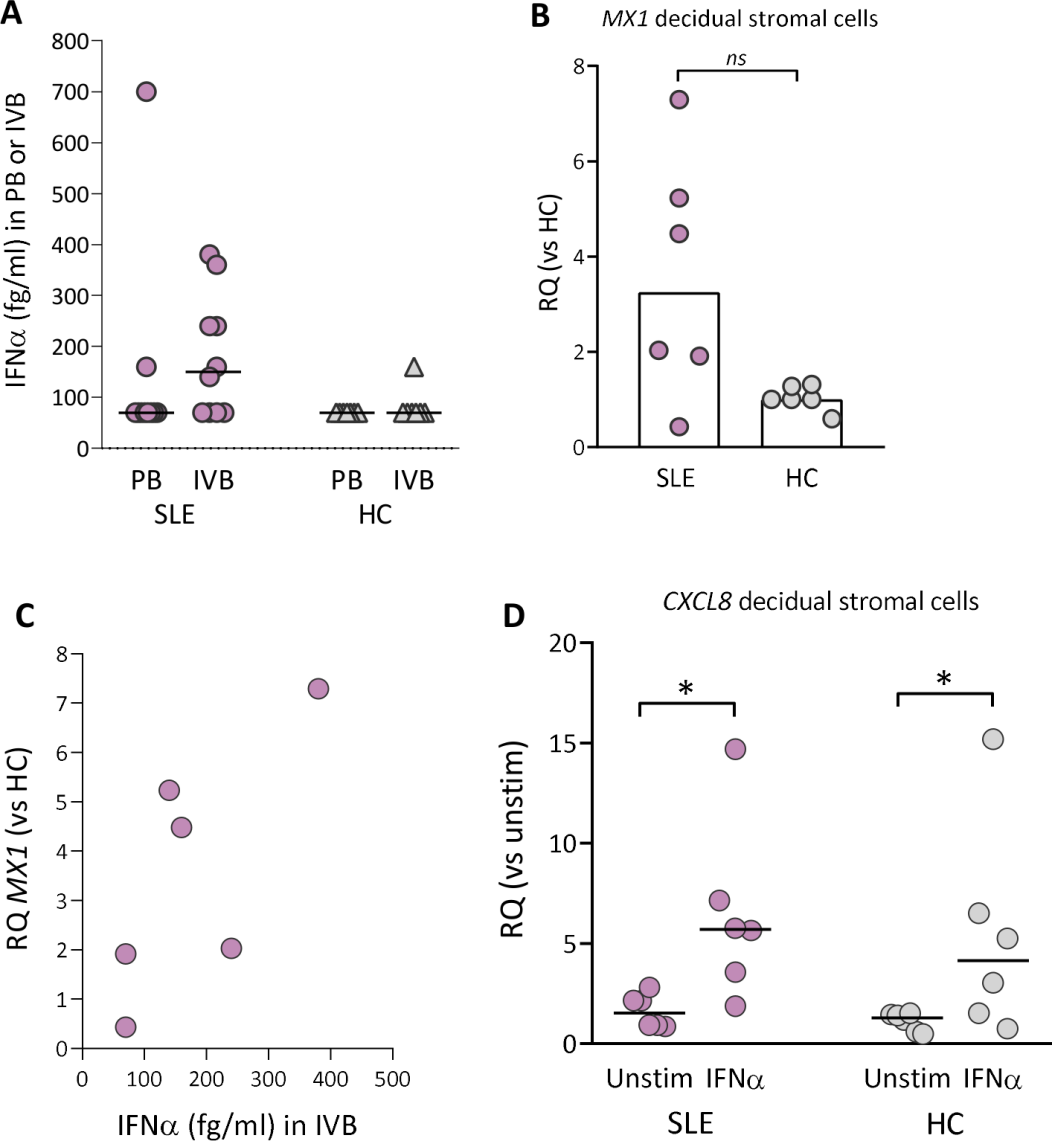
IFNα protein levels in peripheral or intervillous blood and *MX1* gene expression in decidual stromal cells from women with SLE and healthy controls. (A) IFNα protein levels in plasma obtained from peripheral or intervillous blood at delivery in women with SLE and healthy controls (HCs). (B) Quantitative PCR analysis of *MX1* gene expression in decidual stromal cells from women with SLE and HC. (C) Correlation between IFNα protein levels in plasma from intervillous blood and *MX1* gene expression in decidual stromal cells from women with SLE. (D) Quantitative PCR analysis of *CXCL8* gene expression in decidual stromal cells from women with SLE and HC stimulated with IFNα (1 ng/mL) or not. Relative quantification (RQ) was calculated using the average *CXCL8* expression in unstimulated HC controls as a reference. Horizontal bars in A and D indicate medians, scatter plot bars in B display median and each symbol represents one individual. (B) Mann-Whitney U test and (D) *p≤0.05 Wilcoxon matched-pairs signed rank test.

About 50%–75% of adult patients with SLE have a marked overexpression of type I IFN-inducible genes.^15^ In SLE pregnancy, IFNα protein was detectable in 2 out of 10 peripheral blood samples and in 6 out of 10 intervillous blood samples (figure 4A). In healthy pregnancy, IFNα was detectable in one out of seven intervillous blood samples and undetectable in peripheral blood samples. Subgroup statistical analysis of IFNα levels with respect to APO could not be performed due to low number of individuals (online supplemental figure 7A, B). The IFNα-responsive gene *MX1* was upregulated in decidual stromal cells from five out of six women with SLE compared with healthy controls (p=0.06; figure 4B). Even with a low number of subjects, there was linear relationship between IFNα levels in intervillous blood and *MX1* expression in decidual stromal cells in SLE pregnancy (r=0.75 and p=0.1; figure 4C). Finally, IFNα stimulation in vitro significantly upregulated the *CXCL8* gene expression in decidual stromal cells from both SLE and healthy pregnancy (figure 4D). Collectively, these data demonstrate that decidual stromal cells from both SLE and healthy pregnancy respond to IFNα with an increased ability to secrete CXCL8 in vitro, but detectable IFNα protein production and *CXCL8* gene expression in vivo were only found in SLE pregnancy.

### Histological chorioamnionitis in SLE pregnancy

**Figure 5:**
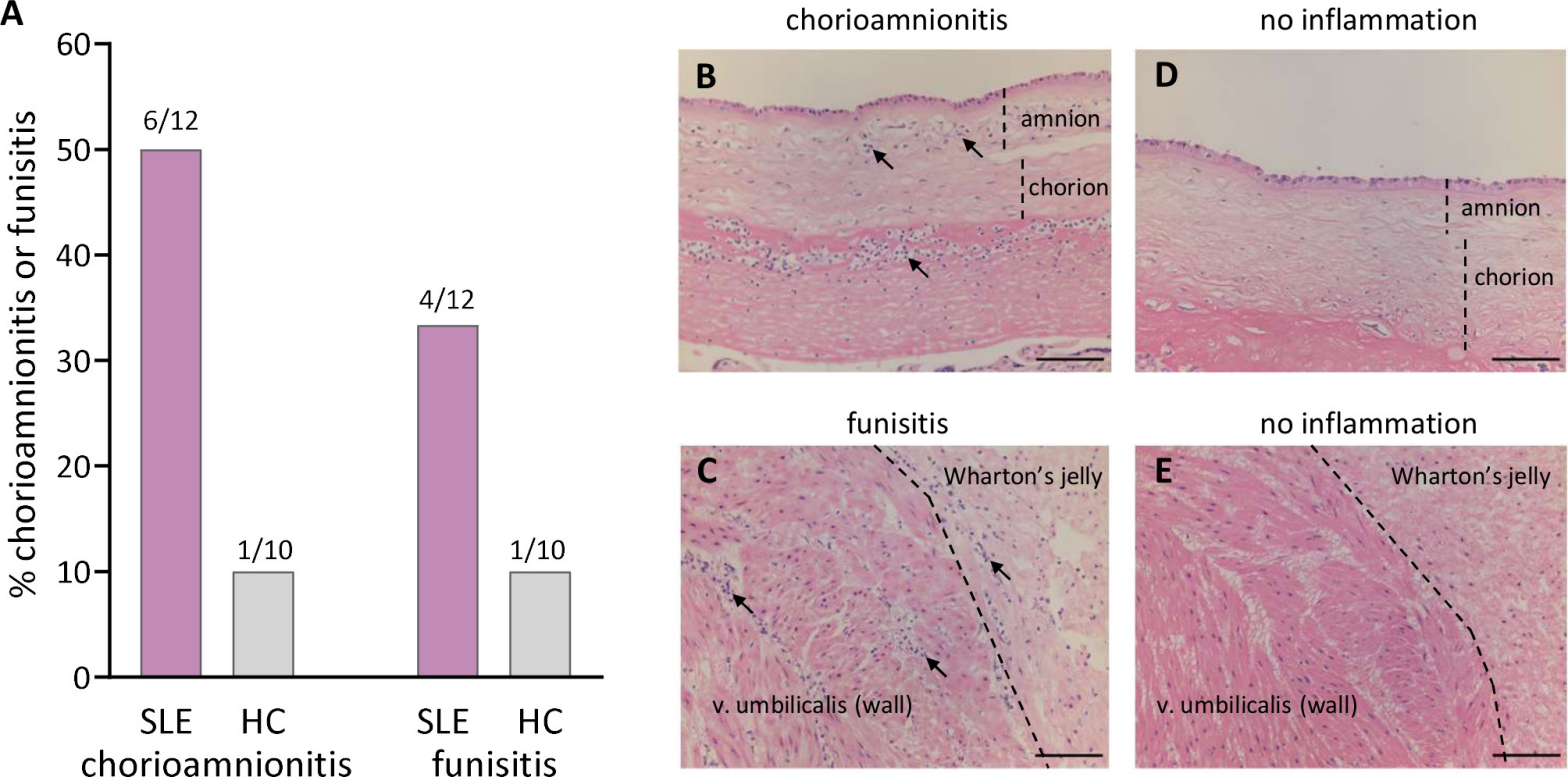
Histological neutrophil inflammation in placenta and umbilical cord. Presence of acute chorioamnionitis or funisitis (A) in the placental fetal membranes and umbilical cord among women with SLE and healthy controls (HCs). Representative histological images of chorioamnionitis (B), funisitis (C), no chorioamnionitis (D) or no funisitis (E) from two patients with SLE. Scale bar=100 µm. Neutrophilic granulocytes indicated by solid arrows.

As shown in figure 5A, B, histological acute chorioamnionitis, infiltration of neutrophils in the fetal chorionic and amniotic membranes and in the subchorionic fibrinoid layer, was diagnosed in 6 out of 12 women with SLE and in 1 out of 10 healthy controls (p=0.07). None of the neutrophil-related variables, including proportions and activation of LDG and neutrophil-recruiting and activating chemokines, were significantly related to chorioamnionitis (data not shown). Moreover, IFNα was measured in five of the SLE patients who had chorioamnionitis and two of these had detectable IFNα in intervillous blood (Fisher’s exact test p=0.5). Funisitis, infiltration of neutrophilic granulocytes in the umbilical vessels and Wharton’s jelly, was diagnosed in 4 out of 12 women with SLE and in 1 out of 10 healthy controls (figure 5A, C). Histological images without chorioamnionitis and funisitis are shown in figure 5D, E, respectively.

## Discussion

Women with SLE are at increased risk of adverse pregnancy outcomes, but the underlying immunological mechanisms are largely unknown. The recognised association of neutrophil activation with SLE pathogenesis in non-pregnant patients prompted us to examine the presence and activation of both NDGs and LDGs in peripheral and intervillous blood in SLE compared with healthy pregnancy. In the present study, we report augmented proportions of LDG in peripheral blood and increased activation of LDG in both peripheral and intervillous blood in SLE compared with healthy pregnancy. Furthermore, we demonstrate a neutrophil-attracting chemokine gradient in intervillous compared with peripheral blood in women with SLE but not in healthy controls. Another novel finding was the increased presence of histological chorioamnionitis, neutrophil infiltration in the placental fetal membranes, in SLE compared with healthy pregnancy.

Neutropenia affects about 20% of adult patients with SLE,^25 26^ but it has also been reported that patients with SLE may exhibit elevated circulating neutrophil numbers compared with controls due to prednisone treatment.^27^ In the present study, we found no differences in the total number of granulocytes in either peripheral or intervillous blood in SLE compared with healthy pregnancy at the time of delivery. The total granulocyte count in both groups was higher compared with counts of neutrophils reported in the referred studies, which may depend on the state of active labour at sampling. Indeed, there is an increase in neutrophil blood count as pregnancy advances.^28^

The concept of different neutrophil subpopulations has attracted attention in recent years.^29–31^ The LDG subset was discovered in non-pregnant SLE patients^10^ and has since been demonstrated in a number of inflammatory conditions and may at least in part consist of activated degranulated neutrophils with increased buoyancy.^32^ In line with this, we here report augmented proportions and increased activation of LDG in peripheral blood from women with SLE compared with healthy women at delivery. Also in intervillous blood, the LDG subset from women with SLE displayed a more activated phenotype relative to healthy pregnancy, but there was no difference in the proportion of LDG between the two groups. In fact, LDG constituted more than 50% of the mononuclear cells in intervillous blood from both SLE and healthy pregnancies. The reason for this can only be speculated on, but the active labour process itself may lead to increased granulocyte activation and degranulation in the placenta.^33 34^ Whether activated LDG are associated with placental pathology in SLE pregnancy remains to be examined, but SLE LDG may be retained in microvasculature networks^35^ and are associated with vascular inflammation.^36 37^ Furthermore, SLE LDG spontaneously secrete proinflammatory cytokines and the neutrophil-attracting and activating chemokine CXCL8^12^ and spontaneously form NETs that may lead to endothelial apoptosis and vascular dysfunction.^11^

A previous study has reported increased numbers of neutrophils and more NETs visualised by immunofluorescence in the placental intervillous space of women with SLE compared with controls,^9^ but how neutrophils are recruited to this site is unknown. We here show that CXCL8 and CXCL1 levels were higher in intervillous compared with peripheral blood in SLE but not in healthy pregnancy, which suggests that there exists a neutrophil-attracting chemokine gradient between intervillous and peripheral blood in women with SLE. Stromal cells are central for recruitment and modulation of immune cells through their secretion of chemokines and cytokines.^38–40^ We found that the *CXCL8* and *CXCL1* gene expression was upregulated in decidual stromal cells from some of the SLE pregnancies compared with controls, which corresponded to the interindividual variation in protein levels of these two chemokines in intervillous blood. Thus, our data indicate that there is an increased ability of neutrophil recruitment to the placenta in SLE pregnancy and that decidual stromal cells could be a cellular source of neutrophil-attracting chemokines during pregnancy.

The genetic IFN type I signature is present in about 50%–75% of adult patients with SLE.^15^ We here show that IFNα protein was detectable in intervillous blood from 60% of the SLE pregnancies and that the IFN-responsive gene *MX1* was upregulated in decidual stromal cells from five out of six women with SLE. We also found that decidual stromal cells from both SLE and healthy pregnancy upregulated the *CXCL8* gene expression in response to IFNα stimulation in vitro. Collectively, our data suggest that increased IFNα protein levels are present in the placenta in a subgroup of SLE pregnancies where it may act as an endogenous adjuvant that triggers decidual stromal cells to secrete CXCL8. This could lead to increased neutrophil recruitment to this organ in these women.

Histological examination of placentas from SLE pregnancies is not generally performed in routine clinical practice; therefore, information regarding presence of acute inflammation in placentas from SLE compared with healthy pregnancies is sparse. It has been reported that women with SLE or antiphospholipid syndrome have more placental vascular lesions and a trend for increased lesions of chronic inflammation compared with healthy controls.^41^ In the present study, acute histological chorioamnionitis, infiltration of neutrophilic granulocytes in the fetal chorioamniotic membranes, was diagnosed in 60% of the SLE pregnancies and in 10% of the healthy pregnancies. Neutrophils are normally not present in these membranes and migrate from the maternal-derived decidua. Accordingly, chorioamnionitis is a maternal inflammatory response, and the infiltrating neutrophils are of maternal origin.^13 42 43^ In the present study, we cannot rule out the possibility that a bacterial infection is the cause of histological acute chorioamnionitis, but others have shown that histological chorioamnionitis in term placentas is often caused by non-infectious inflammation.^13 14^ A fetal inflammatory response, funisitis, with neutrophil infiltration in the umbilical cord vessels and surrounding tissue was also over-represented in SLE compared with healthy pregnancy. Despite a low prevalence of adverse pregnancy outcomes and low disease activity in the present cohort, women with SLE still presented with neutrophil inflammation in the placenta on both the maternal and the fetal side. A larger cohort of patients is needed to study effects of immunosuppressive treatment and clinical disease activity in relation to placenta pathology and immunology.

The use of a clearly defined cohort of nulliparous women with SLE and healthy controls should be considered a strength of this study. Second, for the first time, we examined neutrophil inflammation and activation in peripheral and intervillous blood in SLE pregnancy. Third, histological examination of placentas, which is not performed in routine clinical practice and therefore rarely examined in previous studies, was performed in both groups. Limitations of this study are the small study size, comprising 12 patients with SLE and 10 healthy controls, and that not all immunological assays were applied to all samples. Yet, we still demonstrate clear immunological differences with increased neutrophil activation and inflammation in blood and placenta in women with SLE relative to healthy women.

In summary, the role of the placenta as an immunological organ in SLE pregnancy is incompletely understood. We here report increased proportions of activated LDGs in blood from pregnant women with SLE and a neutrophil-attracting chemokine gradient between intervillous and peripheral blood that may potentiate the placental neutrophil inflammation seen in SLE pregnancies. A possible scenario is that an increased recruitment of neutrophils to the placenta during pregnancy progression may contribute to decreased placental function and increased risk of pregnancy complications in women with SLE.

## Data Availability

All data relevant to the study are included in the article or uploaded as supplementary information.
