# Supplementary material for: Activated low-density granulocytes in peripheral and intervillous blood and neutrophil inflammation in placentas from SLE pregnancies
Source: Lupus Sci Med. 2021 Mar 7;8(1):e000463. doi: 10.1136/lupus-2020-000463 (PMC7942245; doi:10.1136/lupus-2020-000463)
Supplement: Supplementary data [file lupus-2020-000463supp001.pdf]

Supplementary Figure 1

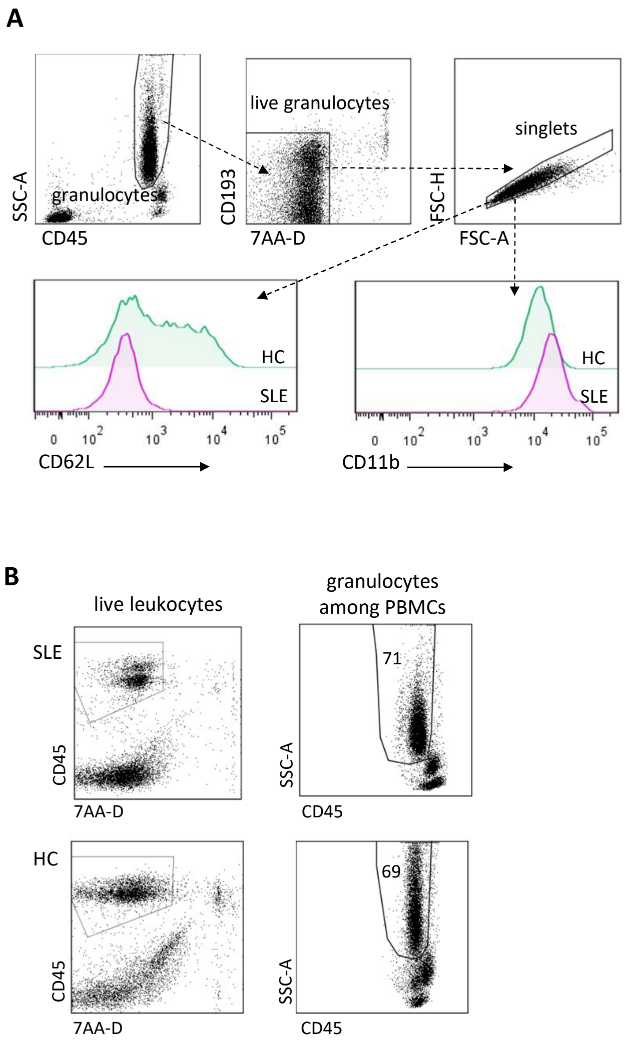

Supplementary Figure 1. (A) Representative gating strategy for live singlet high-density granulocytes (HDG) in intervillous blood, and representative histograms that depict surface expression of CD62L and CD11b within the HDG subset in one woman with SLE and one healthy control (HC). **(B)** Representative gating strategy for live singlet low-density granulocytes (LDG) among $CD45^{+}$ blood mononuclear cells in intervillous blood from one woman with SLE and one HC.

Supplementary Figure 2

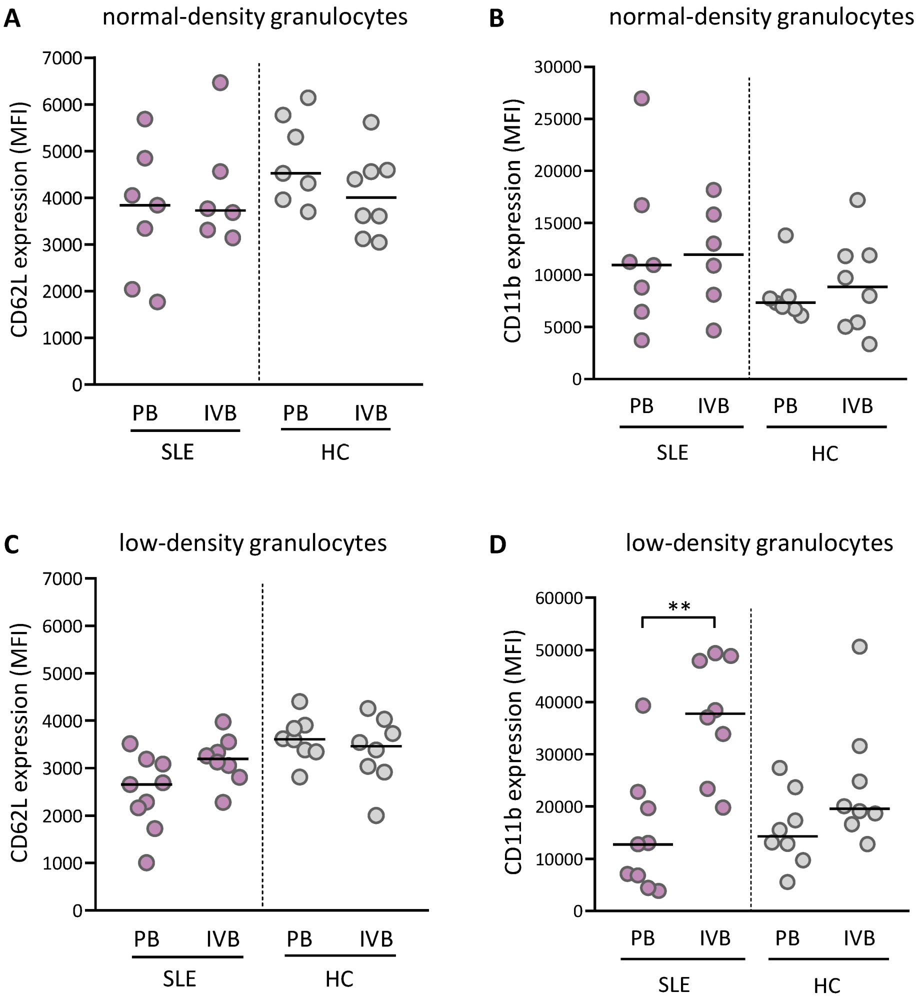

Supplementary Figure 2. Surface expression of CD62L and CD11b on normal-density granulocytes **(A-B)** and on low-density granulocytes **(C-D)** in peripheral relative to intervillous blood in women with SLE or in healthy controls (HC). **p≤0.01 Mann-Whitney U test

Supplementary Figure 3

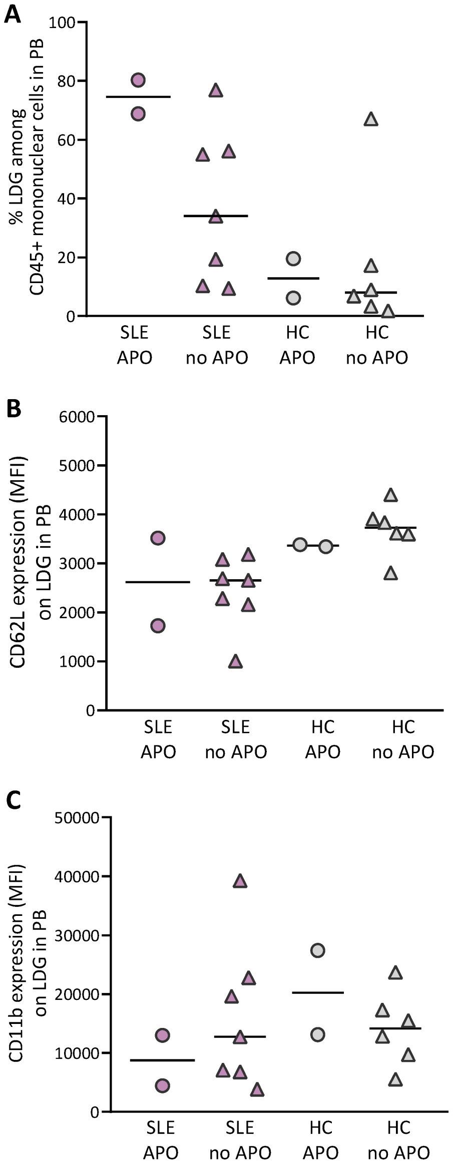

Supplementary Figure 3. (A) Percentage of LDG, **(B)** CD62L expression on LDG and **(C)** CD11b expression on LDG in peripheral blood from SLE patients or healthy controls with (SLE APO and HC APO) or without (SLE no APO and HC no APO) adverse pregnancy outcomes.

Supplementary Figure 4

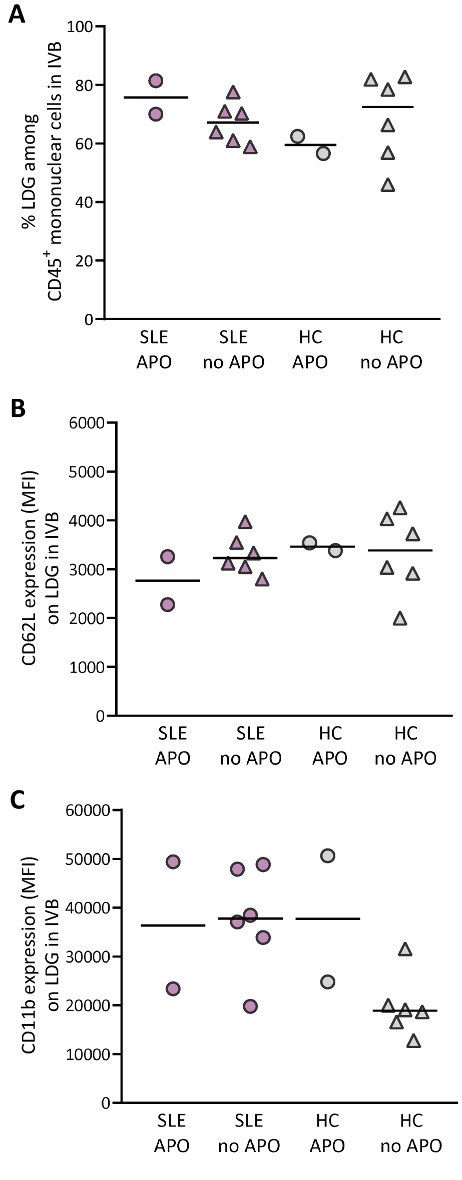

Supplementary Figure 4. (A) Percentage of LDG, **(B)** CD62L expression on LDG and **(C)** CD11b expression on LDG in intervillous blood from SLE patients or healthy controls with (SLE APO and HC APO) or without (SLE no APO and HC no APO) adverse pregnancy outcomes.

Supplementary Figure 5

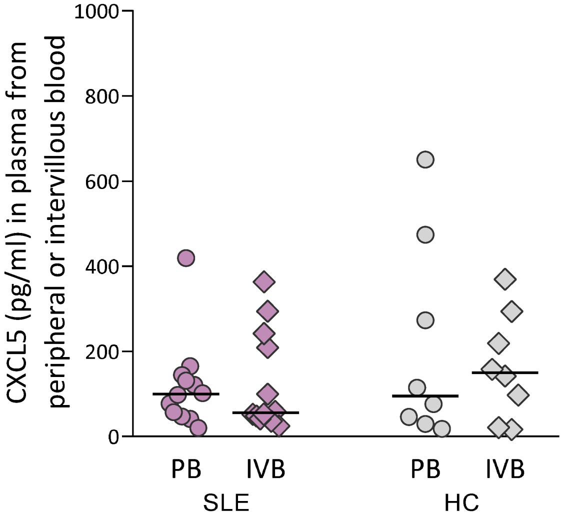

Supplementary Figure 5. Levels of CXCL5 in plasma from peripheral or intervillous blood at delivery in SLE and healthy pregnancies.

Supplementary Figure 6

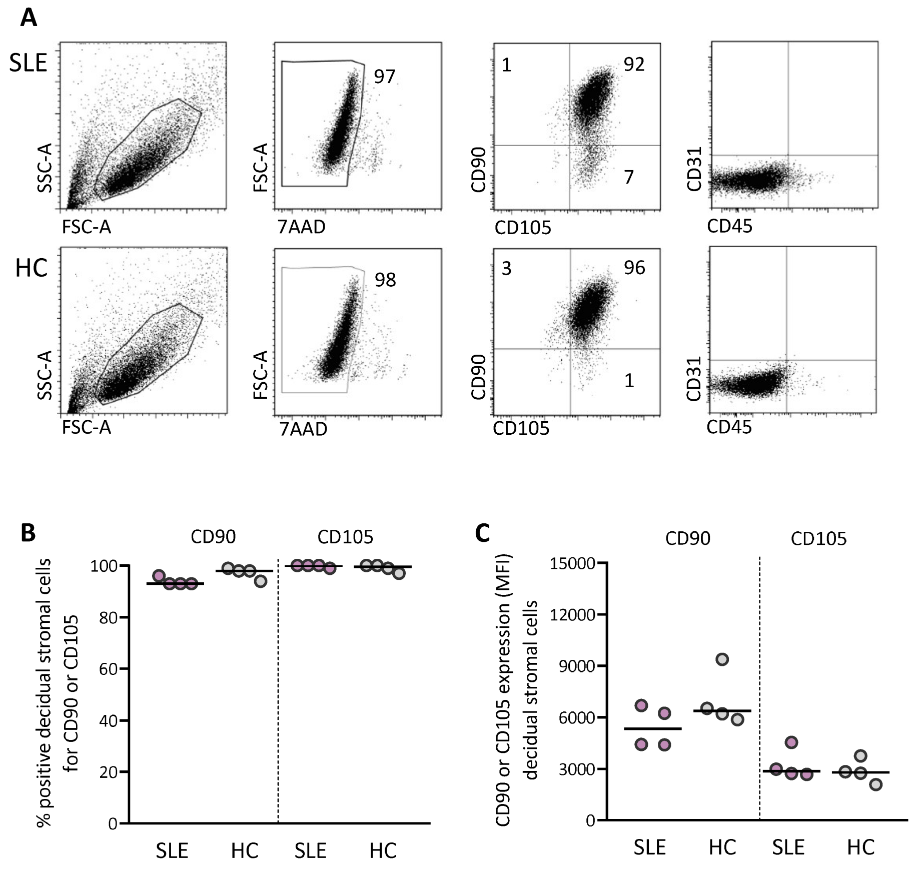

Supplementary Figure 6. (A) Representative flow cytometry dot plots depicting the gating strategy for expression of CD90, CD105, CD31 and CD45 on decidual stromal cells from one women with SLE and from one healthy control (HC). **(B)** The proportion of decidual stromal cells that express the characteristic stroma cell markers CD90 and CD105 from women with SLE and HC, and **(C)** the mean fluorescence intensity of CD90 and CD105.

Supplementary Figure 7

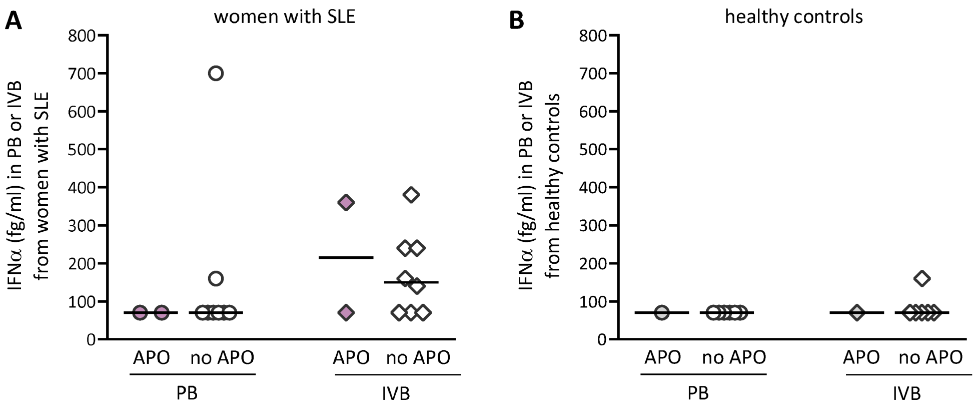

Supplementary Figure 7. IFNα protein plasma levels in peripheral or intervillous blood from **(A)** SLE patients or **(B)** healthy controls with (APO) or without (no APO) adverse pregnancy outcomes.
